# Sri Lanka’s early success in the containment of COVID-19 through its rapid response: Clinical & epidemiological evidence from the initial case series

**DOI:** 10.1371/journal.pone.0255394

**Published:** 2021-07-29

**Authors:** Carukshi Arambepola, Nuwan Darshana Wickramasinghe, Surangi Jayakody, Sumudu Avanthi Hewage, Ananda Wijewickrema, Nalika Gunawardena, Sapumal Dhanapala, Shamini Prathapan

**Affiliations:** 1 Department of Community Medicine, Faculty of Medicine, University of Colombo, Colombo, Sri Lanka; 2 Department of Community Medicine, Faculty of Medicine and Allied Sciences, Rajarata University of Sri Lanka, Anuradhapura, Sri Lanka; 3 Department of Community Medicine, Faculty of Medical Sciences, University of Sri Jayewardenepura, Nugegoda, Sri Lanka; 4 Ministry of Health and Indigenous Medical Services, Colombo, Sri Lanka; 5 National Institute of Infectious Diseases, Angoda, Sri Lanka; 6 World Health Organization, Country Office, Colombo, Sri Lanka; Ohio State University Wexner Medical Center Department of Surgery, UNITED STATES

## Abstract

**Background:**

Despite the rising global burden, Sri Lanka reported a relatively low caseload and mortality (13 deaths as of 20 October 2020) for COVID-19. This warrants exploration of the clinical and epidemiological characteristics of the case series during the initial passive case detection phase in Sri Lanka, in order to understand the success in containment of the disease for more than nine months in the country through its initial early and rapid pandemic response.

**Methods:**

A retrospective study was conducted using secondary data (hospital records and investigation reports) extracted from all laboratory-confirmed patients admitted to the three main state-sector hospitals in Sri Lanka from 11 March to 7 April 2020. Clinical outcomes were categorized as mild, severe and critical, as per the international classification. Kruskal-Wallis H, Mann Whitney U and Fisher’s exact tests compared differences between the variables.

**Results:**

The case series comprised 146 live discharges and six deaths. Majority were males (63.2%); mean age of 40.9 (SD = 17.9); and symptomatic (72.4%). Sixty-one (40.1%) had co-morbidities, the commonest being diabetes (20.4%) and hypertension (17.8%). Mild (93.4%), severe (2.6%) and critical (4.0%) disease outcomes were noted. Disease severity was significantly higher with older age (p = 0.037), co-morbidities (p = 0.026); and higher white-cell (p = 0.045) and lymphocyte (p = 0.043) counts; but not with being symptomatic (p = 0.683). The commonest symptoms were fever (62.5%), dry cough (48.0%) and sore throat (26.9%). The median duration (IQR) was 3.0 (1.0–5.0) and 18.0 (15.0–29.5) days, respectively before and during hospitalization.

**Conclusions:**

In contrast to high-risk countries, the younger age, milder disease and low mortality observed in local patients during the initial phase of the pandemic, reflect the early success in containment of the pandemic in Sri Lanka. However, once the disease becomes severe, the factors determining disease fatality remain the same as in other countries. This highlights the value of establishing strong public health systems and disease surveillance in a country, which could provide long-term effects on disease control.

## Introduction

Undoubtedly, COVID-19 pandemic has become the greatest calamity that the humankind has faced since World War II. Since its emergence in Asia late last year, the virus has spread to every continent except Antarctica. The giant nations like USA, Europe and India have come under siege, with majority countries reporting their second and third waves of community spread [1]. Consequences of the disease have been apparent not only related to the disease burden but also to its socio-economic impact. Worst of all, despite having many strategies in place for prevention and control of the disease, the mortality rates following COVID-19 are still on the rise [2]. Mortality rates range within 3–5% [3]. During last month, Asia reported the second highest number of cases and deaths [2]. However, quite ironically, Sri Lanka, an island nation in the Indian Ocean reported one of the lowest number of deaths in Asia (13 deaths along with 5811 cases as of 20 October 2020), which had stabilized around not more than five deaths per day until late March 2021 [3]. This signifies a unique situation in contrast to the rising number of cases as well as deaths during this period within its closest neighbour, India. This would warrant further exploration of the situation in Sri Lanka.

Sri Lanka is a popular tourist destination in the world, with a high turnover of tourists especially from Europe and South Asia. It is also an economic hub especially for the Chinese workforce occupying main cities and the Port in Sri Lanka. Sri Lanka reported its first COVID-19 case on 27 January 2020—a tourist from Hubei Province, China [4]. The local index case emerged almost after 1½ months on 11 March 2020—a tour guide with contact history of an Italian tourist group [4]. Since its first local case, Sri Lanka’s response to the COVID-19 pandemic has been swift, decisive and well-coordinated. As first steps, the country implemented compulsory 14-day quarantine for returnees from abroad at designated quarantine centres and established capacity for laboratory testing for early identification of patients with COVID-19 [5]. Along with it, a passive case detection phase was declared which was characterized by testing of all suspected patients who fit into the case definition admitted to isolation centres of designated hospitals [6], such as symptomatic persons with an overseas travel history or a close contact history with a case. In addition, persons irrespective of whether having symptoms or not who showed an epidemiological link to a case found in a quarantine centre or at home, were also tested [6]. Once a laboratory confirmed diagnosis of COVID-19 was made, all of them were exclusively admitted to designated state sector hospitals, while their close contacts were actively traced via an efficient disease surveillance system maintained by the Epidemiology Unit of the Ministry of Health Sri Lanka [4] and admitted to quarantine centres managed by the state sector. Within a short time period, passive case detection along with contact surveillance was in operation throughout the country (approximately 1000 tests per day). Within the first month, the number of cases was increasing gradually, mostly attributed to an influx of infected Sri Lankan expatriates returning from Italy, Middle East and the UK. By early April, three clusters confined to very low numbers were reported outside the quarantine centres, following which the case detection strategy was revised to embark on active case surveillance since 7 April 2020, which included mandatory testing of all overseas returnees as well as close contacts and other high-risk groups [7]. Until November 2020, there has not been any evidence of community transmission other than only a few clusters of cases, and with less than five deaths per day recorded due to COVID-19 [4].

Although the first cases of COVID-19 were reported around the same time across countries in the world including in South Asia, evolution of the disease with time within each country has been quite different, indicating differences in the disease epidemiology, even within the same geographical region. Clinical presentation and the course of illness are expected to differ from country to country depending on the climate, virus strain, population structure and socio-economic background and, also due to country response [1]. In exploring for such differences, there is a substantial amount of published literature on case presentation and clinical course of COVID-19 disease, nevertheless most of this evidence comes from China or Europe, where there are many epidemiologically relevant differences especially compared to the South Asian region. The information related to South Asia on COVID-19 pandemic is scarce, thus we report the disease epidemiology, clinical presentation, course, treatment and outcomes of the series of laboratory confirmed patients in the initial passive case detection phase in Sri Lanka, in order to understand the successful containment of the disease for a sustained period in the country. This evidence will point to key lessons learnt on the evolution of COVID-19 in relation to the pandemic response.

## Materials and methods

### Study design and participants

A retrospective study was conducted among COVID-19 patients who were admitted during the initial passive case detection phase of the epidemic (11 March—7 April 2020), to the three main state sector hospitals designated for treating COVID-19 in Sri Lanka. These hospitals comprised the National Institute of Infectious Diseases (NIID), Colombo East Base Hospital (CEBH) and Base Hospital Welikanda. All admissions had their diagnosis confirmed by real time Reverse Transcription-Polymerase Chain Reaction (RT-PCR) performed on nasopharyngeal throat swab, nasal swab or sputum samples, and analysed in three state laboratories in the country (Medical Research Institute, Centre for Dengue Research at University of Sri Jayewardenepura and NIID laboratory). Patients were included consecutively to the study, with no exclusions considered.

### Data collection

Ethical clearance for this study was obtained from the Ethics Review Committee of the Faculty of Medicine, University of Colombo, Sri Lanka (EC/20/EM01). Administrative clearance for data collection was obtained from the Director General of Health Services in Sri Lanka and from all institutional heads.

All available and extractable clinical and epidemiological data of the patients at the time were extracted manually from the paper-based medical records maintained at the hospital, laboratory and radiological investigation reports, and daily clinical status reports specifically maintained on COVID-19 by the respective hospitals. In the case of patients transferred from another institution, information was also obtained from the documents pertaining to his/her last hospital admission or stay in quarantine centres. For this purpose, a data extraction sheet was developed based on the World Health Organization (WHO) Technical Guidelines on Surveillance [5] and anecdotally reported evidence on COVID-19 from all over the world. Consequently, all relevant data recorded throughout the hospital stay until discharge/death were perused to obtain information on socio-demographic characteristics of the patients, contact history of the infection, clinical presentation and the course, treatment and the clinical outcome. Results of the full blood count performed on every patient at various time points of the clinical course, and other investigations conducted such as serum electrolytes, serum proteins, coagulation profile, culture and chest x-ray were also recorded. Computer tomography (CT) scan of chest had not been performed on any patient. Five experienced researchers performed the extraction from available records, while ensuring maximum quality of the secondary data collected.

### Operational definitions

‘Clinical course’ of a patient was defined from the onset of the first clinical symptom (or from the laboratory confirmation of COVID-19 in asymptomatic patients) up to their hospital discharge/death. The day on which real time RT-PCR test became positive for the first time was considered the ‘first test positive’ in the clinical course; and ‘last test negative’ as the day on which the second of the two consecutive real time RT-PCR tests became negative. ‘Co-morbidities’ were defined by any chronic non-communicable or metabolic disease coexisting with COVID-19, as per recorded in the patients’ medical records. ‘Clinical outcomes’ of COVID-19 were categorized as 1) mild disease: having non-pneumonia or mild pneumonia; 2) severe disease: having dyspnoea, respiratory frequency ≥30 per minute, blood oxygen saturation ≤93%, PaO_2_/FiO_2_ ratio <300, and/or lung infiltrates >50% within 24–48 hours; and 3) critical disease: having respiratory failure, septic shock, and/or multiple organ dysfunction/failure [8].

### Statistical analysis

Basic socio-demographic characteristics of the patients, exposure histories, clinical course, investigations performed, treatment modalities and outcomes were described using median (inter-quartile-range (IQR)), mean (standard deviation (SD)) and frequency distributions. Mann Whitney U test and Kruskal-Wallis H test were performed to compare variables by sex, age groups, clinical outcomes and admission status. Fisher’s exact test was used to compare differences of categorical variables between subgroups.

## Results

The number of COVID-19 patients who were admitted to hospital during 11 March—7 April 2020 was 152. They comprised 6 deaths (3.9%) and 146 hospital discharges (96.1%). The last date of discharge of them was 23 May. The majority (n = 95; 62.5%) were treated at NIID. During this study period, Sri Lanka had stage 2 COVID-19 transmission with only sporadic cases reported. All patients included in this study were either the returnees from abroad or the local cases, in whom the source could be epidemiologically linked. Furthermore, no clusters of cases were reported from elderly care homes, residential homes or from any institutions.

### Demographic and epidemiological characteristics of patients

Demographic and epidemiological characteristics of the patients are summarised in Table 1. Approximately two thirds (63.2%) of the patients were males. The mean age was 40.9 years (SD = 17.9), ranging from three months to 85 years. All were Sri Lankans except two (1.3%) nationals of India and France. Seventy patients (46.1%) reported a recent history of foreign travel, while there were eight healthcare workers (5.3%) following work-related exposure to infection. Exposure histories were retrieved from the medical records and were based on the self-reported exposure status of whether a patient could precisely recall an exposure to a confirmed COVID-19 patient. The responses varied from direct contact with a COVID-19 patient (n = 43; 28.3%) or a foreigner (n = 11; 7.2%), to the majority not being aware of an exact contact (n = 84; 55.3%). Sixty-one (40.1%) patients had at least one co-morbidity, the commonest being diabetes mellitus (n = 31; 20.4%), hypertension (n = 27; 17.8%), dyslipidaemia (n = 11; 7.2%) and bronchial asthma (n = 8; 5.3%).

**Table 1:** Clinical outcome of patients by patient and disease characteristics.

Characteristics		Clinical outcome			p value ^1^
		Mild	Severe	Critical	
Sex	Male (n = 96)	87	3	6	0.13
	Female (n = 56)	55	1	0	
Age group (years)	< 18 (n = 16)	16	0	0	0.04
	18–59 (n = 114)	109	2	3	
	≥ 60 (n = 22)	17	2	3	
Health-care worker	Yes (n = 8)	7	1	0	0.22
	No (n = 144)	135	3	6	
Recent overseas travel	Yes (n = 70)	65	2	3	1.00
	No (n = 82)	77	2	3	
Precise exposure to a COVID-19 case identified by the patient^2^	Yes (n = 43)	42	1	0	0.33
	No (n = 109)	100	3	6	
Co-morbidities	None (n = 91)	88	2	1	0.03
	One (n = 35)	33	1	1	
	Two or more (n = 26)	21	1	4	
Symptom status	Symptomatic (n = 110)	101	3	6	0.68
	Pre-symptomatic (n = 23)	22	1	0	
	Asymptomatic (n = 19)	19	0	0	

^1^ p values were calculated using Fisher’s exact test at 0.05 significance level.

^2^ Exposure history based on the self-reported details of whether a patient could exactly report his/her exposure to a confirmed COVID-19.

### Clinical outcomes

According to the severity of infection, 93.4% (n = 142) had mild disease, 2.6% (n = 4) had severe disease, and 4.0% (n = 6) had critical disease. The severity of disease was significantly associated with older age (p = 0.037) and higher number of co-morbidities (p = 0.026) (Table 1).

### Clinical course

The duration of clinical course is presented by age, sex and clinical outcome (Table 2). The median (IQR) duration of illness prior to hospitalization was 3.0 days (1.0–5.0) and that during the hospital stay was 18.0 days (15.0–29.5). The median (IQR) duration of the overall clinical course was 20.0 days (17.0–31.0) days. There was no significant difference in relation to the clinical course in the sub-group analysis (p<0.05).

**Table 2:** Duration of the clinical course of patients by patient and disease characteristics.

Characteristics		Before admission		Hospital stay		Total clinical course	
		Duration^1^	p ^2^	Duration^1^	p ^2^	Duration^1^	p^2^
Sex	Male (n = 96)	3.0 (2.0–4.5)	0.38	18.0 (15.0–29.5)	0.74	20.0 (17.0–30.5)	0.59
	Female (n = 56)	2.5 (1.0–5.0)		18.5 (15.5–29.5)		21.0 (17.5–32.5)	
Age group (years)	< 18 (n = 16)	1.0 (1.0–4.0)	0.25	16.0 (13.0–19.5)	0.07	17.0 (14.0–20.0)	0.03
	18–59 (n = 114)	3.0 (1.0–4.0)		18.5 (16.0–33.0)		20.5 (18.0–31.0)	
	≥ 60 (n = 22)	3.0 (1.0–6.0)		20.5 (16.0–33.0)		24.5 (17.0–34.0)	
Clinical outcome	Mild (n = 142)	3.0 (1.0–4.0)	0.13	18.5 (16.0–31.0)	0.09	20.0 (17.0–32.0)	0.24
	Severe (n = 4)	4.5 (2.5–6.5)		20.5 (16.5–29.0)		21.5 (17.0–30.5)	
	Critical (n = 6)	5.0 (3.0–7.0)		13.0 (9.0–18.0)		18.0 (13.0–20.0)	
Health worker	Yes (n = 8)	3.0 (1.5–4.5)	0.83	17.5 (12.5–29.0)	0.45	23.5 (16.5–29.5)	0.97
	No (n = 144)	3.0 (1.0–5.0)		18.0 (16.0–29.5)		20.0 (17.0–31.0)	
Recent overseas travel	Yes (n = 70)	3.0 (1.0–4.0)	0.28	19.0 (18.0–27.0)	0.09	20.0 (19.0–27.0)	0.56
	No (n = 82)	3.0 (1.0–5.0)		17.5 (14.0–31.0)		21.0 (16.0–33.0)	
Co-morbidities	None (n = 91)	3.0 (1.0–4.0)	0.15	18.0 (15.0–28.0)	0.89	20.0 (17.0–30.0)	0.43
	One (n = 35)	3.0 (1.0–5.0)		19.0 (16.0–28.0)		23.0 (17.0–34.0)	
	≥ Two (n = 26)	4.0 (2.0–7.0)		18.0 (15.0–39.0)		22.5 (18.0–35.0)	
Symptom status	Symptomatic (n = 110)	4.0 (2.0–5.0)	<0.01	20.0 (15.0–33.0)	0.03	23.0 (18.0–35.0)	0.01
	Pre-symptomatic (n = 23)	1.0 (1.0–3.0)		18.0 (16.0–33.0)		20.0 (17.0–24.0)	
	Asymptomatic (n = 19)	1.0 (1.0–3.0)		16.0 (14.0–18.0)		17.0 (14.0–20.0)	
**Total (N = 152)**		**3.0 (1.0–5.0)**		**18.0 (15–29.5)**		**20.0 (17–31)**	

^1^ Duration given as median (IQR).

^2^ p values were calculated using Mann-Whitney U test and Kruskal-Wallis H test as appropriate at 0.05 significance level.

### Clinical symptoms

Majority (n = 110; 72.4%) were symptomatic at the diagnosis. Of the remaining (n = 42), 23 (54.8%) were pre-symptomatic, while 19 (45.2%) were asymptomatic. All the critical patients were symptomatic at diagnosis, while all asymptomatic patients had mild disease. The symptomatic, pre-symptomatic and asymptomatic groups did not differ by the clinical outcomes (p = 0.683); however, they had significantly different durations of clinical course (p<0.05).

Fever was the commonest symptom (n = 95; 62.5%) followed by dry cough (n = 73; 48.0%), sore throat (n = 41; 26.9%), body aches/myalgia (n = 37; 24.3%) headache (n = 34; 22.4%) and diarrhoea (n = 30; 19.7%). Among the fever patients, 90.5% (n = 86) had fever on or before hospital admission, while it was either newly developed or persistent only in 46.3% (n = 44). Despite being a common symptom affecting 33 patients, diarrhoea was present only in 30.0% (n = 9) on or before admission, while 80.0% (n = 24) patients had diarrhoea after admission. Dry cough, sore throat and body aches/myalgia were more prominent prior to hospitalization than later in the course, in contrast to gastrointestinal symptoms, which were more prominent after admission.

Similarly, further analysis on the timing of each symptom revealed that patients had developed fever and dry cough somewhat early in the clinical course (on average within the first two days), while the onset of gastrointestinal symptoms were relatively late (on average in 5–7 days), coinciding with late presentations during hospital stay.

With regards to different combinations of symptoms shown on or before hospital admission, fever most commonly combined with dry cough (30.0%) and also with body aches/myalgia (20.0% and sore throat (15.0%). A similar pattern was noted when these combinations were considered throughout the clinical course. In addition, 18.0% of the patients had fever and diarrhoea during the clinical course. Fever, dry cough, and body aches/myalgia were the commonest symptom triad present on admission (n = 12, 12.0%). The combination of body aches/myalgia and dry cough without fever (14.0%) was also a noteworthy common presentation.

### Biochemical parameters

Results of the laboratory investigations of patients by different sub-groups are summarised in Table 3. The median (IQR) duration of first positive and last negative PCR test results of all patients was 3.0 days (1.0–6.0) and 19.0 (16.0–30.0) days, respectively. The median (IQR) white blood cell (WBC) count was 7.5X10^9^/l (5.9–9.3X10^9^/l). The differential count results showed a median (IQR) lymphocyte count of 34.4% (25.5%-41.5%). There were statistically significant differences in WBC count (p = 0.045) and lymphocyte count (p = 0.043) by clinical outcome.

**Table 3:** Results of the laboratory investigations of the participants by patient and disease characteristics.

Characteristic		First positive real time RT-PCR days		Last negative real time RT-PCR days		Total WBC count		Lymphocyte count	
						10^9^/l		%	
		Median (IQR)	p ^1^	Median (IQR)	p ^1^	Median (IQR)	p ^1^	Median (IQR)	p ^1^
Sex	Male	3.0 (1.0–5.5)	0.51	19.0 (16.0–30.0)	0.95	7.4 (6.0–9.1)	0.78	33.6 (23.7–41.8)	0.44
		n = 96		n = 96		n = 75		n = 72	
	Female	2.0 (1.0–6.5)		20.0 (16.0–29.0)		8.0 (5.6–9.7)		34.8 (28.7–40.9)	
		n = 56		n = 56		n = 48		n = 44	
Age group (years)	< 18	1.0 (1.0–4.0)	0.07	16.5 (13.5–20.5)	0.07	9.1 (5.8–10.7)	0.59	36.4 (32.4–40.3)	0.94
		n = 16		n = 16		n = 7		n = 5	
	18–59	3.0 (1.0–6.0)		19.0 (16.0–31.0)		7.4 (6.0–9.1)		34.5 (26.0–41.5)	
		n = 114		n = 111		n = 97		n = 92	
	≥ 60	4.0 (2.0–8.0)		23.0 (18.0–30.0)		7.7 (5.9–10.5)		32.9 (25.2–43.7)	
		n = 22		n = 19		n = 19		n = 19	
Health care worker	Yes	5.5 (3.5–8.5)	0.07	24.0 (19.0–30.0)	0.48	6.2 (5.1–10.8)	0.52	38.9 (25.7–42.7)	0.72
		n = 8		n = 8		n = 7		n = 7	
	No	3.0 (1.0–6.0)		19.0 (16.0–30.0)		7.6 (6.0–9.2)		34.4 (25.3–41.3)	
		n = 144		n = 138		n = 116		n = 109	
Recent overseas travel	Yes	3.0 (1.0–4.0)	0.09	19.0 (17.0–27.0)	0.99	6.5 (5.7–8.1)	<0.01	34.3 (26.9–40.1)	0.58
		n = 70		n = 67		n = 60		n = 58	
	No	4.0 (1.0–8.0)		21.0 (15.0–33.0)		8.4 (6.6–10.5)		34.5 (25.3–42.7)	
		n = 82		n = 79		n = 63		n = 58	
Co-morbidities	None	3.0 (1.0–6.0)	0.1	19.0 (15.0–28.0)	0.37	7.4 (6.0–9.1)	0.47	34.7 (23.6–40.4)	0.64
		n = 91		n = 90		n = 68		n = 62	
	One	3.0 (1.0–5.0)		21.0 (16.0–28.0)		7.0 (5.9–9.0)		35.2 (29.3–42.7)	
		n = 35		n = 34		n = 33		n = 33	
	≥Two	4.5 (2.0–8.0)		22.0 (18.0–35.0)		8.3 (6.4–10.5)		30.0 (25.7–41.0)	
		n = 26		n = 22		n = 22		n = 21	
Clinical outcome	Mild	3.0 (1.0–6.0)	0.14	19.0 (16.0–30.0)	0.38	7.8 (6.1–9.4)	0.05	35.1 (27.1–41.8)	0.04
		n = 142		n = 142		n = 115		n = 108	
	Severe	5.0 (3.0–6.5)		26.0 (21.0–31.0)		5.4 (4.8–5.8)		29.8 (19.1–36.6)	
		n = 4		n = 4		n = 4		n = 4	
	Critical	5.5 (4.0–8.0)		-		6.6 (6.1–8.7)		18.6 (12.4–27.1)	
		n = 6				n = 4		n = 4	
Symptom status	Symptomatic	4.0 (3.0–8.0)	<0.01	22.0 (17.0–35.0)	<0.01	7.1 (5.7–9.0)	0.02	35.2 (25.3–42.1)	0.61
		n = 110		n = 104		n = 91		n = 87	
	Pre-symptomatic	1.0 (1.0–2.0)		17.0 (15.0–23.0)		8.5 (6.6–11.0)		34.3 (24.0–40.1)	
		n = 23		n = 23		n = 20		n = 19	
	Asymptomatic	1.0 (1.0–1.0)		17.0 (13.0–18.0)		8.4 (7.6–9.2)		31.8 (29.6–37.7)	
		n = 19		n = 19		n = 12		n = 10	
**Total**		**3.0 (1.0–6.0)**		**19.0 (16.0–30.0)**		**7.5 (5.9–9.3)**		**34.4 (25.5–41.5)**	
		**N = 152**		**N = 146**		**N = 123**		**N = 116**	

^1^ p value was calculated using Mann-Whitney U test and Kruskal-Wallis H test as appropriate.

### Clinical management

Ninety-four patients (68.1%) were given HCQ, while 39 (28.3%) and six patients (4.3%) were given antibiotics and antiviral drugs (oseltamivir, lopinavir-ritinovir), respectively. Only 15.0% (n = 15) received mild symptomatic treatment, such as steam inhalation and saltwater gargle. According to the medical records, all patients with deteriorating clinical conditions were promptly admitted to the intensive care unit (ICU). They were altogether eight (5.3%) in number, of whom five (62.5%) progressed to critical disease category and succumbed to the disease. Their median (IQR) duration of ICU stay was 8.0 (4.0–9.5) days. The other three patients admitted to the ICU were not ventilated and were discharged alive. One patient in critical disease category died on admission to hospital. According to the medical record information, no patient was deprived of their due treatment, including the ICU care.

### Mortality following COVID-19 infection

Mortality was reported in six out of the 152 patients included in the study, giving an infection fatality rate of 3.9%. All deaths were observed in males of over 40 years. Half of them belonged to the 60 years and above age group. Five of them had co-morbidities (83.3%), such as diabetes, chronic kidney disease, dyslipidaemia and ischaemic heart disease, while all of them had hypertension. All were in critical stage at the time of death and symptomatic at diagnosis.

## Discussion

We report the epidemiological and clinical characteristics of 152 confirmed COVID-19 patients admitted to hospitals designated for treatment in Sri Lanka. To our knowledge, this is the first case series of COVID-19 patients during the initial passive case detection phase in South Asia.

According to our results, majority of the cases were males (63.2%). This is compatible with other cases reported across the globe, including the International Severe Acute Respiratory and Emerging Infections Consortium (ISARIC) reporting on 58% being males among cases from 240 sites across 25 countries predominantly from the developed region [9]. However, a few distinct differences are noted between Sri Lanka and other countries in relation to their age distribution, clinical outcomes and clinical course.

### Age distribution of cases

With regards to the age distribution, patients from Sri Lanka were found to be relatively young (mean age of 40.9 years), while most belonging to 30–59 age category (n = 86; 56.6%), compared to the median age of 71 years (age range = 0 to 104) reported by ISARIC [9] and 63 years reported from New York, USA [10]. This younger age profile of patients could be largely explained by the unique suppression strategies initiated at the early stage of the outbreak in Sri Lanka, which appear to have prevented the rapid spread of the disease within communities, especially among the high-risk categories such as elders who are likely to develop severe outcomes of COVID-19 owing to their higher prevalence of co-morbidities compared to the young.

**Fig 1:**
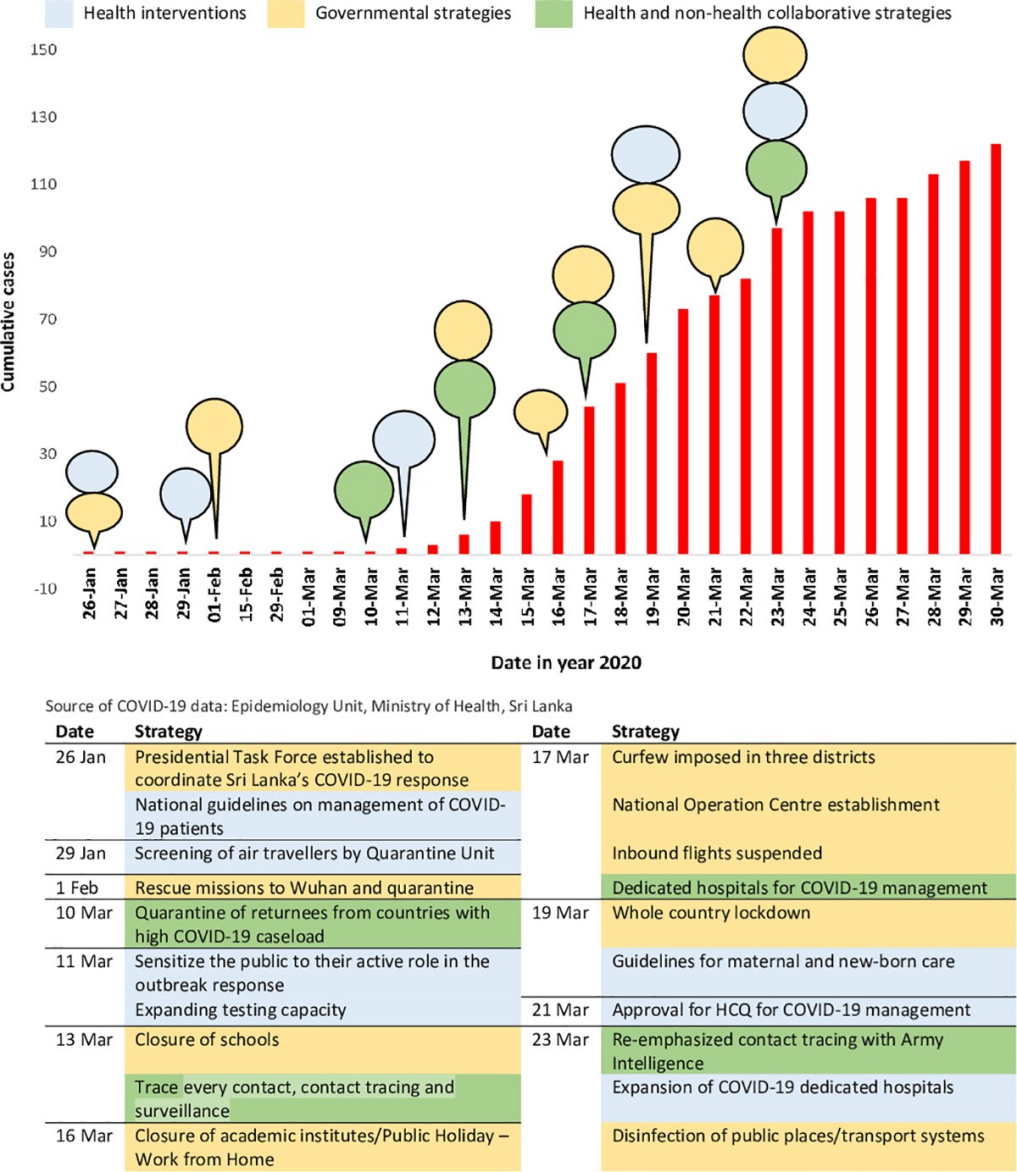
Chronology of the cumulative number of cases and key interventions during the initial phase of COVID-19 in Sri Lanka.

The country achieved this through a cascade of measures implemented by health and non-health authorities in the country [11], such as shutting down of all ports of entry, imposing an island wide lockdown, compulsory 14-day quarantine for returnees from abroad at designated quarantine centers, hospital isolation of confirmed cases, intense contact tracing and household quarantine of their families, and school and university closure (Fig 1).

In this regard, the contribution made by the well-established public health system in Sri Lanka especially its efficient surveillance is noteworthy. It has the capacity to provide door-to-door health services throughout the country via its efficient community-based network of grass root level workers organized into geographically defined 353 health units known as Medical Officer of Health (MOH) areas. Each MOH area covers approximately 60,000 population and is catered by a team of public health staff (public health nursing sisters, public health inspectors and public health midwives, each serving approximately 3,000 population), under the leadership of medical officers of health [12, 13]. This is a unique resource utilized at ground level for active contact tracing and monitoring of home quarantine via home visits paid by the MOH staff to individual households, that no developed country had access to, thus such countries resorting to predominantly secondary prevention, which apparently did not succeed to the same extent as primary prevention. The state military and police extending support in contact tracing and quarantine using the ‘whole-of-government and whole-of-society’ approach was another unique intervention in Sri Lanka [11].

### Severity of the disease

This early public health response could also explain to a large extent the difference observed in the clinical outcomes in Sri Lanka compared to other countries. In Sri Lanka, mild disease was observed in 93.4%; severe disease in only 2.6% and critical disease in 4.0%, compared to mild cases of 81%, followed by severe (14%) and critical patients (5%) using the same disease classification according to Chinese data [8]. In this regard, having ‘early’ lockdowns in Sri Lanka appears to have been effective in protecting especially the high-risk groups, such as older people and those with comorbidities being exposed to the virus, when compared to India which had the first case around the same time as in Sri Lanka, but with a slightly delayed public health response [2]. Also, in comparison with developed countries, having fewer elderly homes or residential care facilities where the disease could spread more easily would have contributed to the higher number of mild cases in Sri Lanka. Furthermore, lessons learnt from China enabled Sri Lanka to conduct prompt training and prepare the healthcare staff with management guidelines, which is reflected by only eight (7.0%) reporting work related exposures to the infection, compared to as much as 63% in Wuhan according to the Chinese Center for Disease Control and Prevention [8]. However, in addition to the early application of stringent measures in the country, genetic factors, virulence of the variants and environmental conduciveness for the virus should also be considered when exploring these differences in disease outcomes. Also, there had been unique features that had substantially contributed towards its containment in Sri Lanka especially when comparing with neighbouring India. Being an island with a population of only 20 million, Sri Lanka is at a better position in restricting movement of people and international migration. Furthermore, other than a few pockets, there are no large slum communities and extremely poor housing conditions with overcrowding as in India, which could lead to rapid spread of the disease. In addition, Sri Lanka possesses a centrally controlled political administrative structure unlike in India where there is much devolution of power to each state, thus difficulty in implementing the preventive measures uniformly.

### Mortality related characteristics

It should be noted that the findings on mortality were not based on a sample, but on all COVID-19 patients detected during passive case detection phase, thus the findings could be extrapolated to the entire population. Even if Sri Lankans differed by their clinical outcomes, once they progress to critical disease, the mortality related characteristics were seen to follow a similar pattern as in other countries. A meta-analysis of seven studies including 1576 confirmed patients from China has indicated that those having hypertension (odds ratio (OR)): 2.36; 95% confidence interval (CI): 1.46, 3.83), respiratory disease (2.46; 1.76, 3.44) and cardiovascular disease (3.42; 1.88, 6.22) and males (2.84; 1.96, 3.74) were more likely to die or shift to critical disease [14]. In concurrence, all except one among the dead had co-morbidities (83.3% with multiple comorbidities) and were in critical disease. In addition, it showed significant associations between worst clinical outcomes and succumbing to the disease. This suggests that, had there been more severe or critical cases, the epidemiological picture in Sri Lanka too would be similar to that of other Asian countries, highlighting the importance of initiating early primary preventive measures for containing the disease successfully at cluster stage, especially in countries with low-resource settings like Sri Lanka, where secondary preventive measures are limited beyond its usual capacity.

Further, mortality observed exclusively among the males and in symptomatic patients compared to most survivors who had mild disease and were symptomatic, is noteworthy. However, these observations are limited by the small number of deaths that took place at the initial phase, thus cannot be cited as prognosis related factors in our study.

### Clinical course

According to our case series, early admission to hospital was noted within three days of the first symptom, yet the hospital stay lasted on average for 18 days, highlighting almost three weeks duration of the clinical course. This was much longer compared to other countries, such as ISARIC [9] and data from China [15]. This observation could have been due to the hospital policy of early admission and not discharging until all symptoms have resolved or two negative test results. With regards to the clinical presentation, fever was the commonest symptom experienced on admission and at any time during the clinical course (62.5%) followed by approximately half experiencing dry cough (48.0%), sore throat (26.9%), body-aches /myalgia and (24.3%). In contrast, shortness of breath was one of the four commonest symptoms reported on admission by ISARIC [9] as well as in meta-analysis including Chinese studies [14]. These findings highlight the importance of developing population-specific case definitions that could capture the local disease epidemiology.

It is noteworthy that all ‘severe’ and ‘critical’ patients were symptomatic at diagnosis, while all asymptomatic and pre-symptomatic patients had ‘mild’ disease and seemed to clear the virus sooner than the symptomatic patients. This further reflects the successful outbreak response in Sri Lanka, where the case detection process in Sri Lanka during the initial phase of the outbreak managed to identify the contacts of the diagnosed patients early, so that a substantial proportion of them were asymptomatic at the laboratory diagnosis and thereby more likely to have milder disease forms.

With regards to the infection status of patients, the median (IQR) lymphocyte count was significantly higher in patients with mild disease, in comparison to those who had severe or critical disease. This finding is compatible with Time Lymphocyte Model (TLM) developed in Wuhan, China using cases of COVID-19, where the first point (TLM-1) which was taken after 10–12 days of symptom onset, patients with lymphocyte >20% are classified as moderate type with full recovery; and <20% are classified as severe type with adverse outcomes [16].

## Conclusions & recommendations

Compared to other countries currently at increased risk of COVID-19, the profile of Sri Lankan patients in the initial passive case detection phase is compatible with patients’ sex distribution, clinical symptoms and disease course. In contrast, the younger age of patients, milder disease outcome and low mortality observed in local patients may reflect the early success that had sustained for more than nine months following the rapid preventive and curative health sector response to the pandemic in Sri Lanka. However, once severe disease is established, the factors that determined fatality remain the same as in other countries.

The findings suggest that rapid response to COVID-19 is of use in containing the disease quite early in the epidemic, which could lead to lasting effects on the disease epidemiology in countries, as evident in this study from Sri Lanka. In this regard, well-established public health systems as well as case detection surveillance networks are equally important at national level for achieving long-term benefits on novel diseases such as COVID-19.
